# Opening Up Editorial Boards

**DOI:** 10.1128/spectrum.04937-22

**Published:** 2022-12-21

**Authors:** Christina A. Cuomo

**Affiliations:** a Infectious Disease and Microbiome Program, Broad Institute of MIT and Harvard, Cambridge, Massachusetts, USA

**Keywords:** editorial board, open application

## EDITORIAL

Over the past year and a half since the relaunch, *Microbiology Spectrum* has rapidly grown as a wide scope journal for rigorous microbiological science and is now the largest journal with the ASM portfolio. During this initial period, we have prioritized aligning growth to core values that advance open science. A key facet we included from the start of the relaunch was to utilize an open process for building our editorial board. While we also recruit editors through direct invitations, which is the typical approach at many journals, we primarily rely on self-nominations by microbiologists to serve as editors for *Spectrum*. This conduit of open applications to join an editorial board is fundamentally inclusive, as it avoids biases such as personal networks that can influence who can participate at the editorial level. Practically, the open application process resulted in a large pool of potential editors during our growth phase and was crucial to assemble a board of engaged editors who subscribe to the journal mission and bring expertise across microbiological research. This open search also enabled us to form an editorial board with diverse representation, including race and ethnicity, geography of home institution, and the type of workplaces across academic, non-profit, health care, and industry settings. We view this diversity as essential for the collective work of the journal editors to support the scientific community.

*Microbiology Spectrum* was re-launched and opened for submissions in April 2021, and that same month, we also opened our application process for candidates to join our open editorial board. The application is streamlined to include the current position of the applicant, demographic information, scientific expertise, and interest in joining our board. In reviewing applications, we evaluated the demonstrated expertise of applicants in leading research in microbiology and prior experience as an editor or reviewer, prioritizing research areas in proportion to the respective number of submissions. We also factored in demographic information, different institution or employer types, and geographic location, to ensure that our editorial board reflected diverse perspectives in microbiology. One challenge we encountered was a substantially lower number of applicants who identified as female, who made up roughly a quarter of the total applicant pool. Based on the large number of applicants, we have been able to come close to gender parity in our editorial board, with 52% men, 46% women, and a small number of non-binary or undisclosed gender. Discussing how to increase applicant diversity not only based on gender identity is a major topic for a working group on diversity and inclusion that we have launched within *Spectrum*.

Enabling anyone around the world to apply and join our editorial board has resulted in a remarkably geographically diverse mix of editors. With our current board of 348 editors, 57% are from outside the United States, and our editors represent microbiology research carried out in 43 countries. Notably, more than half of our editors identify as non-White. While we have substantial numbers of editors who are Asian and Hispanic/Latinx, the number of Black/African American editors is small, and an area we aim to grow with targeted recruitment.

A parallel process has more recently engaged applicants to our open board to serve as reviewing editors at *Spectrum*. Our reviewing editors are an in-house pool of expert reviewers for manuscripts submitted to *Spectrum*, helping to expedite the review process. We currently have 86 reviewing editors on our board, of whom 65% are based outside the United States. This group is also nearly at gender parity, with 54% women, 45% men, and the remainder self-describing their gender. This is a role that can be held by scientists who are early in their career, and we are building a process to provide them feedback and set a path for them to move to handling editor positions in the future.

Our incorporation of an open process for selecting editors has also helped us build an editorial board with expertise across microbiology. The journal currently is organized around 10 subject areas, with the largest editorial representation in virology, bacteriology, antimicrobial resistance, microbiome and clinical microbiology. While we continue to select editors from across these areas, we are now prioritizing applications for journal areas with outsized submission numbers, which we note on our application form.

While the timing of joining an editorial board has many individual factors, there are some common benefits which should be open to everyone. Many of our editors described their interest in joining the board as including support for our publishing model and open science. Journal editing for ASM also supports all the work done by this scientific society. In addition, editors can benefit from seeing behind the scenes of how papers are handled through the review process and can enlist the advice of our senior editors and journal staff to discuss specific questions and deepen their perspectives. Editorial service can be a factor in recognition awards by societies and can also factor into promotions. We feel everyone should have access to such opportunities and have been gratified to see that other journals are adopting practices for opening up editorial boards. At *Spectrum*, we view an open board as central to the mission of the journal to broadly support and engage with microbial scientists around the globe.

